# I knew how it feels but couldn’t save my daughter; testimony of an Ethiopian mother on female genital mutilation/cutting

**DOI:** 10.1186/s12978-017-0434-y

**Published:** 2017-12-01

**Authors:** Yohannes Mehretie Adinew, Beza Tamirat Mekete

**Affiliations:** 1 College of Health sciences and Medicine, Wolaita Sodo University, Sodo, Ethiopia; 2 College of Health sciences and Medicine, Jigjiga University, Jigjiga, Ethiopia

**Keywords:** Female genital mutilation, Female genital cutting, Female circumcision, Genital cutting, FGM/c, Somali, Ethiopia

## Abstract

**Background:**

World Health Organization defines female genital mutilation/cutting as all procedures that involve partial or total removal of the external female genitalia, or other injury to the female genital organs for non-medical reasons. The practice is common in Ethiopia, especially among Somali (99%) ethnic groups. Even though FGM/C is labeled illegal practice according to the revised 2005 Penal Code of the country, the practice is still responsible for misery of many girls in Ethiopia.

**Methods:**

This personal testimony is presented using woman’s own words. Data were collected through in-depth interview with a woman at Gursum health center, Somali regional state, eastern Ethiopia on June 19/2016. The interview was conducted in a private environment and original names were changed to overcome ethical concerns. Informed written consent was obtained from the participant prior to data collection. The interview was audio-taped using a digital voice recorder, later transcribed and translated verbatim from the local language, Amharic to English.

**Results:**

The study participant described a range of experiences she had during her own and her daughter’s circumcision. Three themes emerged from the woman’s description: womanhood, social pressure and stigmatization of uncircumcised women and uncertain future.

**Conclusion:**

Even though the national prevalence may show a decline, FGM/C is still practiced underground. Thus, anti-FGM/C interventions shall take in to account elders influence and incorporate a human rights approach rather than relying merely on the dire health consequences. Further exploration of the determinants of FGM/C on a wider scale is recommended.

## Plain English summary

Female genital mutilation/cutting (FGM/C) is a common deep rooted harmful traditional practice in Ethiopian Somali region. This practice does not seem to end; as the influential members of the community are backing it in secret. The authors sought the opinion of a mother whose daughter had gone through the practice, via face to face interview.

The respondent was asked to share her personal experience as a victim of the practice, attitude of the elderlies (community leaders), fate of someone who refused to have his/her daughter circumcised and what she thinks about the future.

The pain that the participant went through was terrible but not unique to her, as the problem is shared by all women in her community. She blames the elders for sticking to this traditional practice and harassing individuals who refused to comply with their beliefs and values. The respondent didn’t see any hope in the future of girls in her community.

In conclusion, despite the revised 2005 penal code of the country which banned the practice and the “awareness creation campaign” over the years, FGM/C is still behind the misery of many girls. Ultimately this will inform policy makers and guide local stake holders on their next intervention.

## Background

Female genital mutilation/cutting (FGM/C) is defined by World Health Organization (WHO) as all procedures that involve partial or total removal of external female genitalia, or other injury to female genital organs for non-medical reasons. Four types of FGM/C are known. Type I is the partial or total removal of the clitoris. Type II is the partial or total removal of the clitoris and the labia minora. Type III is narrowing of the vaginal opening through creation of a covering seal. The seal is formed by cutting and repositioning labia minora and/or labia majora, and often with stitching. Type IV consists of all other harmful procedures to female genitalia like pricking, piercing, incising, scraping and cauterization for non-medical reason [1].

FGM/C, a violation of girls’ and women’s rights, can cause long-term physical and emotional damage [2]. It may lead to the death of mothers and babies during childbirth [3, 4]. It may also cause many short and long-term complications. The immediate health complications include hemorrhage, shock and infection. The long term health risks are chronic pain, keloid formation, primary infertility and psychological consequences [5–7].

FGM/C is a common practice in many societies in sub-Saharan Africa [8, 9]. In Ethiopia, though the age at which FGM/C is performed varies among different ethnic groups commonly it is done throughout childhood. However in Somali region girls get circumcised mostly after they celebrated their fifth birth day [10].

FGM/C has garnered broad attention in Ethiopia; and the practice has decreased over the past decade, dropping from 74% in 2005 to 65% in 2016. The decline is more remarkable among the young; 24% fall during same period. This prominent reduction among younger women is partly because of under reporting the practice to avoid legal consequences [10], as the revised 2005 Penal Code [11] made FGM/C illegal. This show, after the campaigns against the practice, people have not changed their attitudes and practices around FGM/C. This resistance may be due to the dominant role and influence men have in the society. Men’s preference of circumcised woman for marriage, which is often cited as one of the central motives for maintaining the practice [12] is an indication that the elderlies want the practice to continue through generations [13].

Nevertheless the revised 2005 Penal Code banned FGM/C; the practice continues to contribute to major health problems of many girls in Ethiopia (65%). particularly female circumcision is still most prevalent among Somali (99%) ethnic group. Type 3, infibulation, is the most practiced type in the region with a prevalence of 73% [10]. Thus the aim of this testimony was to hear in one woman’s own words, what the experience of FGM/C was for the mother of a recently circumcised daughter who suffered excessive blood loss following the procedure.

## Methods

This personal testimony is presented using woman’s own words. The study was conducted at Gursum health center, Somali regional state, eastern Ethiopia on June 19/2016. The study participant was a purposively selected mother of a recently circumcised daughter who had also undergone the practice. Data were collected through in-depth interview at a silent place chosen by the participant. The interview was audio-taped using a digital voice recorder. The main topics covered were her personal experience as a woman that had undergone the practice, attitude of the elderlies (community leaders), fate of someone who refused to have his/her daughter circumcised and what she thinks about the future. The interview was transcribed and translated verbatim from the local language, Amharic to English. The transcript and translated version of the document was cross checked with the original interview by an experienced sociologist familiar with Somali culture.

## Results

### Demographic characteristics of the study participant

The study participant was a thirty two years old Somali house wife with no formal education. She has got three children; the youngest being the only daughter.

### Case of Fathma

A Bajaj, three-wheel vehicle, rushed to Gursum health center, Somali regional state. Inside the car were a six years old girl and her Mother. Ellihan (*name changed*) presented with severe bleeding from the cut and was exhausted by blood loss, pain and crying. The health center team acted instantly to help her. As Ellihan was receiving treatment inside emergency room, I sat with her mom, Fathma (*name changed*) to ask what has happened to her daughter. She started to cry “*I knew how it feels but couldn’t save my daughter, I feel very sorry for my poor baby*”. Meanwhile a nurse came out to assure the mother that her daughter was getting all the necessary treatments and would be ok. As I continued to comfort Ellihan’s mother, Ellihan’s crying came to an end from inside. After a while a male nurse in charge of the health center came with the good news, “*the bleeding has stopped and the child is sleeping well*”; only then the mother took a long breath and thanked the lord. Once she saw her child in emergency room she stopped crying and became calm.

### Findings from the in-depth interview

In-depth interview was used to learn from the woman about her experiences. The study participant described a range of experiences she had during her own and her daughter’s circumcision. Three themes emerged from the woman’s description: womanhood, social pressure and stigmatization of uncircumcised women and uncertain future.

### Womanhood

Since I was aware of the reason for her daughter’s bleeding, I asked her if she had also gone through the practice. She said “*I had a fresh memory of it albeit that took place a couple of decades ago*”. Fathma started to narrate it with deep sorrow “*I remember everything they did to me as if it was yesterday and I can never forget it. I was only ten when I underwent the circumcision. The pain I experienced always compels me not to forget that day. One cursed morning; my mom told me that it was the right time for me to join womanhood and for that I had to be circumcised. The next day one woman, a self-taught circumciser, came to our home and discussed the importance of the procedure with my family.”*

While Fathma was narrating how they held her to the ground and cut her genitalia, she became very emotional and cried but continued to tell her story “*our neighbors were also in our home to see my suffering. The woman (circumciser) ordered them to tie my legs and hold me to the ground firmly; they complied. I cried as the woman approached me with the blade. One hand immediately covered my eyes and I couldn’t see anymore. I couldn’t move my body as I was tied and held to the ground by adults. But I cried out loud and called my mom for help, it didn’t happen; the woman cut and stitched my body. What was worse was not the cutting, but the stitching. Words can’t express the pain I went through. When they released my eyes I immediately searched for my mom but couldn’t find her anywhere in the house, after a while she came and comforted me that it all would be okay and the wound would heal soon. But I could see from her eyes that she was also crying. When I was with my mom, that evil woman (circumciser) came and told me to sleep and not to wash my body to facilitate the healing. At that time when I inspect my surroundings*, *my leg and the floor was covered by blood. It really seemed there had been a red flood, shame on their heartlessness*.”

Fathma kept silent for a while and continued after taking a deep breath “*I didn’t know if it was because I cried a lot, the pain or they held me tight to the ground, I was so tired and fell asleep where they left me. When I woke up my mom was waiting for me sitting on the ground. When she saw me awake she hugged me tight and said, ‘It is over, now you are a woman that every boy wants to marry and our community respects’. She said many more things to help me overcome the pain. However, I resume crying in mom’s arm as I couldn’t tolerate the pain, it burned so much. Watching me in that kind of pain made my mom cry with me, like what I did today with my daughter, Ellihan. Today, I really understand my mom; I was accusing her, my whole life, for not protecting me from the circumciser. But after twenty years, I found myself in her shoes. Despite the pain and injury I suffered that took a long time to heal, I couldn’t help my daughter escape this reckless procedure.”*

After a couple of minutes she held her breath and continued “*I remember what I felt during my first sexual intercourse. Even though it may be enjoyable for many, for the women in my community it is different. The day before my marriage my mother in-law took me to a nearby clinic to open the stiches made during the circumcision and checked for my virginity. I had sex with my husband a day after the suturing was cut. Even though my genitalia was bleeding and I had wound, I have to go through what other women did; nothing was different for me as it was common to every woman in my community. I shouted and cried loud as I was unable to withstand the pain. It seems easy to talk about once it has passed. I even remember a woman who fainted during sex due to the overwhelming pain. Delivery was also the other death sentence I survived. If it had not been in a health facility I would not be with you today. The nurse told me I survived the impossible when she discharged me from the hospital six days after my admission.”* Fathma cried again.

### Social pressure and stigmatization of uncircumcised women

Fathma is still very angry at her community particularly to the elders for not stopping this harmful traditional practice after years of awareness creation programs and community dialogues. “*Our community is very conservative and resistant to change. They say yes and gave their words to government officials and community workers, but they remain the ones who discriminate and outcast those who didn’t undergo through this practice. They will harass you if you don’t respect and follow their beliefs and values. They don’t want to see something different from their way of life. At the beginning I was not willing to have my daughter circumcised, but later I couldn’t withstand the pressure from the elders. They warned me of banning my family from all the social and religious activities if I failed to comply with their order. Then my husband said ‘we have no place to go and can’t go against them, please apologize to the elders and prepare our daughter for the procedure’. It was impossible to resist any more; I felt to be left with no option. I let my daughter suffer this much, at the time she was supposed to play. Look what I did to her; she is now on hospital bed. How can I look in her eyes when she wakes up? I don’t have the guts to stand in front of her. I can never excuse myself”.* Fathima couldn’t talk any longer, and started crying.

### The uncertain future

After a moment of silence, she suddenly stood up and continued talking, pointing her finger at me, *“It is amazing to see still many women encouraging this practice. How on earth can a mother force her child to suffer the same problem she had been through? Even the educated ones are behind this practice, so do you really think that this tragedy will end? I don’t think so, because even to date most men do not want a wife who has not been cut at all. Therefore I cannot see any hope in the future for our little innocent girls.”* When I winded up our conversation I thanked her and visited Ellihan. Ellihan was discharged the next day improved.

## Discussion

Such testimony is enlightening and important to elucidate the experience of a woman using her own words, what it is like to undergo through the experience of FGM/C. The main findings were womanhood, social pressure and stigmatization of uncircumcised women and uncertain future.

Nationally (17.5%) and in Somali region (52.2%) reproductive age women tend to support continuation of the practice while only 11% and 34% men nationally and in Somali region respectively favored it [10]. Most of the consequences of FGM/C appear years after the operation; thus women will not be able to relate the root to its seeds. As a result the positive attitudes towards the custom would outweigh the negative aspects [14, 15]. More importantly this women’s position could be mainly due to the patriarchal nature of the society; where girls’ circumcision is thought to be the rite of passage to womanhood [16] as male prefer to marry a circumcised girl and the community presume the uncircumcised girl as unclean. Therefore mothers will be fearful that their uncut daughters would not be eligible for marriage and are thus compelled to circumcise them [12].

In countries like Ethiopia it is up to the mother to make sure that the children are within the community’s custom as she spent much of her time with them. If circumcision is a precondition for marriage, a mother will get her daughter circumcised. She knew how it feels from her own experience, she might even have suffered a lot but no mother can sit and watch her daughter left unmarried. Even though a daughter may accuse her mother for her circumcision today, there is a high chance that she will also do the same to her daughter to make sure that she does not get less out of life. Because in our society despite her educational level a woman will not get much respect till she found a family. Though men publicly stand against the practice and want it to stop, women shift the blame to them as they are not willing to marry uncircumcised girl. Men want circumcised wife as circumcision is expected to make women more obedient [17] and/or may be as they are less aware of its costs [18]. Even if the controversy needs to be more explored it seems that women are being indirectly forced by the male to favor FGM/C. Thus, if husbands do not expect it women could easily abandon FGM/C [16, 18].

The stigma associated to being uncut can cause more suffering to the woman than if she had had the operation. In most cultures circumcision is performed to protect virginity [19], and make the genitalia hygienic [20] and beautiful [19]. Thus in a society where circumcision is customary uncut woman is considered dirty and promiscuous. As a result she will not get married [21] and will not be allowed to speak at gatherings. Worse still her families may suffer discrimination from the community for failing to follow the custom [22]. Thus, a woman cannot protect her daughter from the procedure even if she wants to [23].

Even if the practice is illegal in Ethiopia, the legal process does not actively punish those who practice FGM/C [24]. This weak enforcement of the law is often attributable to the interference of religious leaders and elders to handle cases through the traditional system [25]. Especially in traditional community like Somali people where the elders are much respected and their say is final, government’s weak commitment towards the fight against FGM/C is a fertile ground for the practice to continue underground [24, 25].

National laws about FGM/C must be appreciated both as legitimate and enforceable by the public. Individuals are unlikely to act against laws if they perceive legal actions are definite [26]. Disseminating prosecution cases could also deter people from practicing FGM/C [27]. However, direct assaults on practices like FGM/C that have cultural implications are certain to fail. Shifts in societal customs come when the influential ones are convinced and realign the public jointly on new ways of thinking about their traditions. Projects that celebrate positive cultural values, appropriate information and discussions regarding human rights can make people abandon harmful practices and help them bring self-directed change [28]. This stresses the consolidation of domestic laws against FGM that seeks the support of elders and religious leaders in the fight to eliminate the practice, and this would be helpful in wining hearts of those who are behind it [18]. Then, even the elders, staunch defenders of FGM/C, can end the practice and create FGM/C free society [29].

## Conclusion

Even though the national prevalence may show a decline, FGM/C is still practiced underground.

Thus, anti-FGM/C interventions shall take in to account elders influence and incorporate a human rights approach rather than relying merely on its dire health consequences. Further exploration of the determinants of FGM/C on a wider scale is recommended.

## Abbreviations

FGC: Female Genital Cutting
FGM: Female Genital Mutilation
WHO: World Health Organization
